# Three Dimensional Human Neuro-Spheroid Model of Alzheimer’s Disease Based on Differentiated Induced Pluripotent Stem Cells

**DOI:** 10.1371/journal.pone.0163072

**Published:** 2016-09-29

**Authors:** Han-Kyu Lee, Clara Velazquez Sanchez, Mei Chen, Peter J. Morin, John M. Wells, Eugene B. Hanlon, Weiming Xia

**Affiliations:** 1 Geriatric Research Education Clinical Center, Edith Nourse Rogers Memorial Veterans Hospital, Bedford, MA, United States of America; 2 Department of Neurology, Rhode Island Hospital and Brown University Warren Alpert Medical School, Providence, RI, United States of America; 3 Department of Pharmacology and Experimental Therapeutics, Boston University School of Medicine, Boston, MA, United States of America; 4 Department of Environmental Health, Harvard T.H. Chan School of Public Health, Boston, MA, United States of America; 5 Medical Research & Development Service, Edith Nourse Rogers Memorial Veterans Hospital, Bedford, MA, United States of America; Cleveland Clinic, UNITED STATES

## Abstract

The testing of candidate drugs to slow progression of Alzheimer’s disease (AD) requires clinical trials that are lengthy and expensive. Efforts to model the biochemical milieu of the AD brain may be greatly facilitated by combining two cutting edge technologies to generate three-dimensional (3D) human neuro-spheroid from induced pluripotent stem cells (iPSC) derived from AD subjects. We created iPSC from blood cells of five AD patients and differentiated them into 3D human neuronal culture. We characterized neuronal markers of our 3D neurons by immunocytochemical staining to validate the differentiation status. To block the generation of pathologic amyloid β peptides (Aβ), the 3D-differentiated AD neurons were treated with inhibitors targeting β-secretase (BACE1) and γ-secretases. As predicted, both BACE1 and γ-secretase inhibitors dramatically decreased Aβ generation in iPSC-derived neural cells derived from all five AD patients, under standard two-dimensional (2D) differentiation conditions. However, BACE1 and γ-secretase inhibitors showed less potency in decreasing Aβ levels in neural cells differentiated under 3D culture conditions. Interestingly, in a single subject AD1, we found that BACE1 inhibitor treatment was not able to significantly reduce Aβ42 levels. To investigate underlying molecular mechanisms, we performed proteomic analysis of 3D AD human neuronal cultures including AD1. Proteomic analysis revealed specific reduction of several proteins that might contribute to a poor inhibition of BACE1 in subject AD1. To our knowledge, this is the first iPSC-differentiated 3D neuro-spheroid model derived from AD patients’ blood. Our results demonstrate that our 3D human neuro-spheroid model can be a physiologically relevant and valid model for testing efficacy of AD drug.

## Introduction

Alzheimer Disease (AD), the most common type of dementia, is characterized by progressive loss of memory and decline of other cognitive abilities that eventually interfere with social functioning. Currently, there are no effective treatments that reverse or substantially slow the progression of AD. The development of therapeutics for AD is plagued by multiple obstacles, including poor translation of pharmacology from cells to humans. Methods are needed to accelerate evaluation of candidate drugs to address the burgeoning prevalence of AD in aging populations.

Developing systems to model AD is challenging due to the extreme complexity of microscopic neuroanatomy and uncertainty regarding key pathogenic steps. AD is primarily characterized by the extracellular deposition of misfolded amyloid-β (Aβ) peptide-containing neuritic plaques and the intracellular formation of neurofibrillary tangles (NFTs), accompanied by neuroinflammation and massive neuronal cell and synapse loss at specific brain regions [1–3]. β-Secretase (BACE1) and γ-secretase are two enzymes that cleave amyloid precursor protein (APP) to generate Aβ. The closest relationship between the Aβ plaques and cognition is found during the early stages of the disease, and this correlation decreases as NFT production and neurodegeneration progress [4–6]. Furthermore, the intensity of cognitive decline seems to correlate with the density of the neuritic plaques [7–9]. As the disease advances to later stages, the relationship between Aβ plaques and cognitive decline becomes weaker [5, 6]. A number of studies have reached similar conclusions concerning the relationship between neocortical NFTs and cognitive impairment. During the initial phase of the syndrome, NFTs are restricted to the entorhinal cortex, progressively spreading to the limbic and medial temporal lobe and correlating with early AD symptoms related to memory [10, 11]. At the end stage, NFTs are more abundant and found in neocortical regions involved in executive function, visual and spatial abilities, and language, skills that are impaired in advanced state AD patients [12, 13]. While it is almost impossible to recapitulate the whole process in vitro, models of AD based on cultured neurons are likely to capture at least some key features of early-stage pathology, especially neuronal generation of Aβ. Yet standard primary neuronal cultures poorly represent the environment of central nervous system since they typically exclude glial cells and the complex 3-dimensional (3D) architecture of cerebral cortex. Modeling the spatial and temporal pathogenic events in a 2 dimensional (2D) cultured cell system seems almost impossible in light of the complexity of 3D neuronal structure enclosed in a human brain.

To improve on cell culture models of disease, interest has turned to 3D cultures. Recently, a brain tissue-like 3D environment was created to cultivate AD pathology development in 3D neuronal culture with some important findings [14]. In traditional 2D cell culture, secreted Aβ species diffuse into the large volume of the cell culture media precluding accumulation of Aβ when the media are routinely changed. In 3D cultures, local Aβ concentrations are apparently high enough to initiate Aβ aggregation and accelerate Aβ deposition. Choi et al reported a deposition of Aβ aggregates in neurons in thin-layer 3D cultures that were differentiated for only 6 weeks [14]. In addition to Aβ aggregation, phosphorylated Tau protein also accumulated, suggesting that both of these processes are accelerated by 3D culture conditions. These results suggest that 3D culture conditions hold great promise for recapitulating Aβ and Tau pathologies and allowing testing of candidate treatments aimed at key pathogenic steps that are not present in 2D cultures. In order to employ these models for AD drug testing, 3D cultures must be carefully assessed for cell behavior, secretase activity, drug penetration, and other factors related to extracellular matrix and the potential for addition of glial cells.

The recently established technology of creating human blood cell-derived induced pluripotent stem cells (iPSC) presents an additional opportunity for improving in vitro models of AD. Several studies demonstrate the importance of this technology. Israel et al. created iPSC lines from two normal subjects, two sporadic AD (sAD1 and sAD2), and two familial AD patients [15]. Human differentiated neurons from two familial AD patients and sAD2 showed very high levels of Aβ1–40, phosphorylated Tau (pTau at Thr 231) and active GSK3β. Importantly, Israel found that levels of Aβ, pTau and active GSK3β can be reduced in these neurons by BACE1 inhibitors, but not γ-secretase inhibitors, indicating a direct relationship between the APP C-terminal fragment (CTF) and GSK3β activation/Tau phosphorylation [15]. Another study compared cultured neurons differentiated from iPSC lines of familial AD patients carrying a mutant PS1 or PS2 gene to those from control, cognitively normal centenarians [16] and found an increased ratio of Aβ42/Aβ40. iPSC-differentiated human neurons have been used to demonstrate accumulation and aggregation of intraneuronal Tau after Tau oligomers were internalized [17]. Similarly, oligomeric Aβ is shown to play a pathological role in inducing endoplasmic reticulum (ER) stress in iPSC-differentiated neurons [18]. These iPSCs were derived from atypical early-onset, autosomal recessive familial AD patients carrying an E693Δ mutation of APP that produces mutant Aβ lacking residue Glu22. When iPSCs were generated from an APP-E693Δ mutant carrier and differentiated into human neurons, Aβ oligomers accumulated in the neurons and induced ER stress, which could be prevented by treatment with a BACE1 inhibitor or docosahexaenoic acid (DHA) [18]. Thus, the iPSC paradigm was used to pinpoint the mechanism of DHA efficacy in a sub-population of subjects whose neurons have high levels of oligomeric Aβ. The iPSC-derived human neurons provide a screening tool for oligomer Aβ quantification and predict whether DHA or BACE1 inhibitors will alter the biology of disease in these AD patients. Such applications demonstrate the feasibility of using iPSC in targeted AD drug discovery and evaluation.

In this study, we combined 3D neuronal cultures and iPSC technology to generate 3D neuro-spheroids from AD patients. To evaluate the utility of this paradigm, we focused on characterizing Aβ generation and drug inhibition in 3D cultures. Using quantitative Mass Spectrometry, we evaluated how drug penetration in 3D cultures differs from that in 2D cultures in which drug diffusion is not limited by compact cellular architecture. Thus, this system may be useful for evaluation of established neuronal features of the AD phenotype and for characterization of the effects of pharmacological agents on these features. These results are significant for future studies employing 3D iPSC-derived cultures to investigate AD pathology and treatment strategies.

## Material and Methods

### Subjects and blood samples

Ante-mortem blood samples were obtained from subjects in a Dementia Special Care Unit at the Bedford VA Hospital Geriatric Research Education and Clinical Center (GRECC). Subjects were hospice patients suffering from advanced dementia. AD subjects were diagnosed with sporadic AD based on early-stage cognitive deficits, age of onset, absence of significant family history, and typical neuroimaging and clinical progression (Table 1). Blood was obtained from the subjects as a part of tissue bank repository as approved by the Bedford VA Hospital Institutional Review Board, and written informed consent was obtained from the participants. Blood was collected in Vacutainer cell tubes (CPT, Becton, Dickinson and Company, Franklin Lakes, NJ) and immediately centrifuged at 1500 ×g for 20 min at room temperature. After centrifugation, the plasma was separated and frozen at -80°C. The peripheral blood mononuclear cell (PBMC) layer was transferred to a 15mL Falcon tube with 10mL sterile PBS and centrifuged at 300 ×g for 10 min at room temperature. The supernatant was discarded and the cell pellet was re-suspended in PMBC medium with 10% DMSO (Invitrogen, Carlsbad, CA) for cryostorage.

**Table 1:** Methods for clinical and/or pathological diagnosis of five AD patients.

AD Case	Age onset of first symptoms	Gender	Family history of early dementia	Neurology/ neuropsych. evaluation	Typical neuroimaging	PET scan	Neuropathological confirmation
**1**	58	M	No	Yes	No	No	Yes
**2**	59	M	No	Yes	Yes	Yes	Yes
**3**	50	F	No	Yes	Yes	Yes	N/A
**4**	85	F	No	Yes	Yes	No	N/A
**5**	48	M	No	Yes	Yes	Yes	N/A

### iPSC generation and expansion

Induced pluripotent stem cells (iPSC) were obtained from frozen or fresh PBMC [19] using the integration-free CytoTune-iPS Sendai Reprogramming Kit (Invitrogen, Carlsbad, CA). This protocol utilizes Sendai virus particles to transduce the four Yamanaka factors [20, 21]. Transduced cells were cultured in mouse embryonic fibroblasts feeder (MEF) cultures and fed with iPSC medium complemented with bFGF (Invitrogen, Carlsbad, CA) until small colonies appeared in about two weeks. The small colonies were maintained for two additional weeks before selection for expansion into individual iPSC lines [22].

### Differentiating iPSC into 2D Neurons

After developing and selecting the iPSC colonies, those colonies were cultured on Geltrex matrix-coated plates with E8 medium (Invitrogen). For differentiation into 2D neurons, the medium was replaced every other day by neural induction medium (Invitrogen) for seven days. On day seven, the neural stem cells were exposed to accutase (Invitrogen) for ~5 min and plated on Geltrex matrix-coated 10 cm plates with a Rock inhibitor (Thiazovivin; 1 uM) (Miltenyi Biotec, San Diego, CA). The following day the medium was replaced by neural expansion medium without Rock inhibitor for 5 days. After 5 passages in neural expansion medium, neural stem cells were plated in neural expansion medium on 6-well plates (2.5–5 x 10^5^ cells) or 8-chamber slides (2.5–5 x10^4^ cells) coated with poly-L-ornithine (Sigma) and Laminin (Life Technology). After two days, the medium was replaced by neuronal differentiation medium (Neurobasal medium with B27 and GlutaMAX) and changed every 3–4 days thereafter.

### Generation of human 3D cortical spheroids from iPSCs

Generation of 3D spheroids from iPSCs was accomplished using a modified protocol [23]. Briefly, the iPSCs in E8 medium with a ROCK inhibitor (Thiazovivin, 1 uM) were transferred into 100 mm ultra-low-attachment plastic plates (Corning, Tewksbury, MA). On the day following formation of the spheroid, the medium was replaced with neural induction medium (Invitrogen) for 6 days. Then the floating spheroids were moved to neural medium (NM) containing Neurobasal, B-27 serum substitute without vitamin A, GlutaMax, penicillin and streptomycin (Invitrogen). The NM was supplemented with 20 ng/ml FGF2 and 20 ng/ml EGF (R&D Systems, Minneapolis, MN). Cells were grown in this medium for 21 days with daily replacement during the first 10 days, and every other day for the subsequent 11 days. To promote differentiation of the neural progenitors into neurons, FGF2 and EGF were replaced with 20 ng/ml BDNF and 20 ng/ml NT3 (Peprotech, Rocky Hill, NJ) starting at day 27. From day 48 onwards, NM without growth factors was used and replaced every four days.

### Drug treatments and media and cell lysate collection

Two dimensional neurons differentiated for 6–8 weeks were treated with inhibitors. Three dimensional neurons differentiated for 9 weeks were evenly distributed into 6-well plates, and the spheroids were treated either with BACE1 inhibitor LY2886721 at 0.1, 0.5 or 1μM (APEXBT, Boston, MA), or γ-Secretase inhibitor Compound E at 0.1, 0.5 or 1μM (EMD Millipore, Billerica, MA). After 2 days of treatment, the media was collected for Aβ 40 and 42 measurement by Enzyme linked immunosorbent assay (ELISA). For quantification of drug exposure, some spheroids were collected after 2 days of treatment and subjected to LC-MS/MS quantification.

### Quantification of Aβ using sandwich ELISA

ELISA was performed to quantify 1–40 and 1–42 using a multiplex kit from Meso Scale Discovery (MSD, Rockville, MD, USA). Briefly, plates were blocked with diluent 35 for 1h at room temperature. Samples were freshly loaded into the wells and incubated with the secondary antibody 6E10 for 2 hr at room temperature. Finally, plates were washed and 150 ul of read buffer was added before reading using the MSD Sector Imager 2400 (MesoScale Discovery, Rockville, MD).

### Immunocytochemistry

To characterize the 2D and 3D neurons derived from iPSCs, cells were immunostained using selected markers. 2D cells were transferred into an 8-chamber well slide (polystyrene vessels culture slides, Falcon) and postfixed with 4% paraformaldehyde (PFA). 3D cultures were also fixed with 4% PFA overnight followed by 30% sucrose solution for 3 days at 4°C. After fixation, 3D neuro-spheroids were transferred into embedding-medium (Tissue-Plus, O.C.T Compound 4585, Fisher HealthCare) and quickly frozen with dry ice. The cells were cut into 10μm thick sections using a cryostat (Leica). Sections were mounted in a superfrost slide and kept on dry ice until immunocytochemical (ICC) staining. Both 2D and 3D cells were blocked in 10% normal goat serum (NGS), 0.1% bovine serum albumin (BSA) and 0.3% Triton X-100 in PBS for 1h at room temperature, followed by overnight incubation at 4°C with primary antibodies. Then, cells were incubated with the appropriate secondary antibodies conjugated with fluorophores, examined and imaged using the confocal microscope (Leica TCS SP5 Confocal Laser Scanning Microscope). All antibodies were commercially available: Tau antibody BT-2, pTau181 antibody AT270, and PAX6 antibody were purchased from ThermoFisher (Waltham, MA); Nestin, Sox1, Sox2, Glial Fibrillary Acidic Protein (GFAP), NeuN, β tubulin III (BT3), and microtubule-associated protein 2 (MAP2) antibodies were purchased from EMD Millipore (Billerica, MA).

### Determination of drug levels by LC-MS/MS

LC-MS/MS method was used to determine if BACE1 or γ-secretase inhibitor were permeable into neuro-spheroids. Spheroids were collected after 2 days of treatment and subjected to LC-MS/MS quantification. The LC-MS/MS system consists of UltiMate 3000 UHPLC automated system coupled with TSQ Quantiva triple quadrupole Mass Spectrometer (Thermo Fisher, Waltham, MA). Samples were prepared by adding 250ul ice cold acetonitrile to each sample vial containing pre-washed 3D neuron cells. Then, each sample was sonicated and vortexed vigorously while keeping the sample cold by immersion into ice between the steps. These steps were repeated until all cells were disrupted. After samples were centrifuged at 12,000 ×g for 10min, the supernatant was aliquoted and diluted with mobile phase A, and then transferred into a HPLC vial for LC-MS/MS analysis. The chromatographic separation was performed on a Kinetex C18 column (50 x 2.1mm, 2.6 um particle size, Phenomenex, Torrance, CA) with mobile phase consisting of water with 0.1% formic acid (mobile phase A) and acetonitrile with 0.1% formic acid (mobile phase B), running a linear gradient from 1 to 90% for 13 min, and then maintaining at 90% for 3 min, back to 1% in 1 min, and maintaining at this proportion for 7 min to equilibrate the column. The flow rate was set to 0.35 mL/min. The MS equipped with an H-ESI source was operated in the positive ionization mode with selected reaction monitoring (SRM). Ion spray voltage was 3.6 kV and ion transfer tube temperature was 325°C. The mass/charge (m/z) ratios monitored were 391.13/269.07, 391.13/287.00 for BACE1 inhibitor LY2886721 and 491.22/221.07, 491.22/249.07 for γ-Secretase inhibitor Compound E. A second transition of each analyte was used for confirmation purposes.

### Proteomic analysis of 3D neurons

#### Sample preparation

100 μg of protein of each neuron sample was reduced with tris(2-carboxyethyl)phosphine (TCEP), alkylated with iodoacetamide and digested with trypsin overnight at 37°C. Labelling of tryptic peptides with Tandem mass tag (TMT) 6-plex reagents (Thermo Fisher) was carried out according to manufacturer’s instructions. The combined TMT labelled samples were cleaned up using C18 tips before analyzing by LC-MS/MS.

#### LC-MS/MS analysis

The HPLC system was coupled to a Q Exactive Orbitrap MS (Thermo Fisher Scientific) with a nano-ES ion source. The TMT labelled peptides were separated by a C18 reverse-phase capillary column. The column was eluted using linear gradients of 2–35% acetonitrile in 0.1% formic acid at a constant flow rate of 300 nL/min for 220 min. The instrument was operated in the positive-ion mode with the ESI spray voltage set at 1.8 kV. The data were acquired in a data-dependent manner using the top 20 most abundant ions for Higher-energy C-trap dissociation fragmentation. The spectral data acquisition was performed using Thermo Xcalibur 3.0.63.

#### Protein identification and quantification

All MS raw data were processed using Proteome Discoverer 1.4 (Thermo Scientific). Data were searched against the human Universal Protein Resource sequence database (UniProt). The search parameters were set as follows: enzyme, trypsin; fixed modification, carbamidomethyl (Cys); variable modification, oxidation (Met), TMT/+ 229D-K, TMT/+ 229D-N Terminal; maximum miss cleavage, 2; 10 ppm precursor tolerance; 0.02 Da fragment ion tolerance; false discovery rate (FDR) at peptide and protein levels, < 0.01; and required peptide length, > 6 amino acids.

## Results

### Generation of Induced Pluripotent Stem Cells from 5 Alzheimer’s Patients

We selected subjects from the GRECC Dementia Special Care Unit at the Bedford VA Hospital based on a clinical diagnosis of AD and the absence of other active medical problems. Some of them underwent brain imaging/PET scan to obtain more accurate diagnosis of AD. Brain tissues from two AD patients were obtained at autopsy, and neuropathological diagnosis of AD was confirmed (Table 1).

Peripheral blood mononuclear cells were prepared within one hour of blood collection and frozen for storage or immediately processed to be transfected with four Yamanaka factors, Oct, Sox2, Klf4, and c-Myc that have been shown to be sufficient for efficient reprogramming. After confirmation of development of human iPSC by karyotyping (data not shown) [22], we characterized these iPSC with immunostaining of sialylated keratan sulfate antigens Tra-1-81 and Tra-1-60, as we previously reported [22]. Since the CytoTune-iPS reprogramming system uses vectors that are non-integrating into the genome, we further confirmed that there was no trace of Sendai viral protein that could be detected by antibody against SeV protein (data not shown). To demonstrate three germ layer differentiation capacities, we tested differentiation in vitro by embryoid body (EB) formation and confirmed the presence of embryonic epitopes (data not shown) using independent antibodies for ectoderm (β III tubulin), mesoderm (smooth muscle actin), and endoderm (α fetoprotein), as we previously reported [22].

### Induction of neural stem cells (NSCs) from iPSCs and generation of human 2D neuronal culture and 3D neuro-spheroids (3DSs) from NSCs

**Fig 1:**
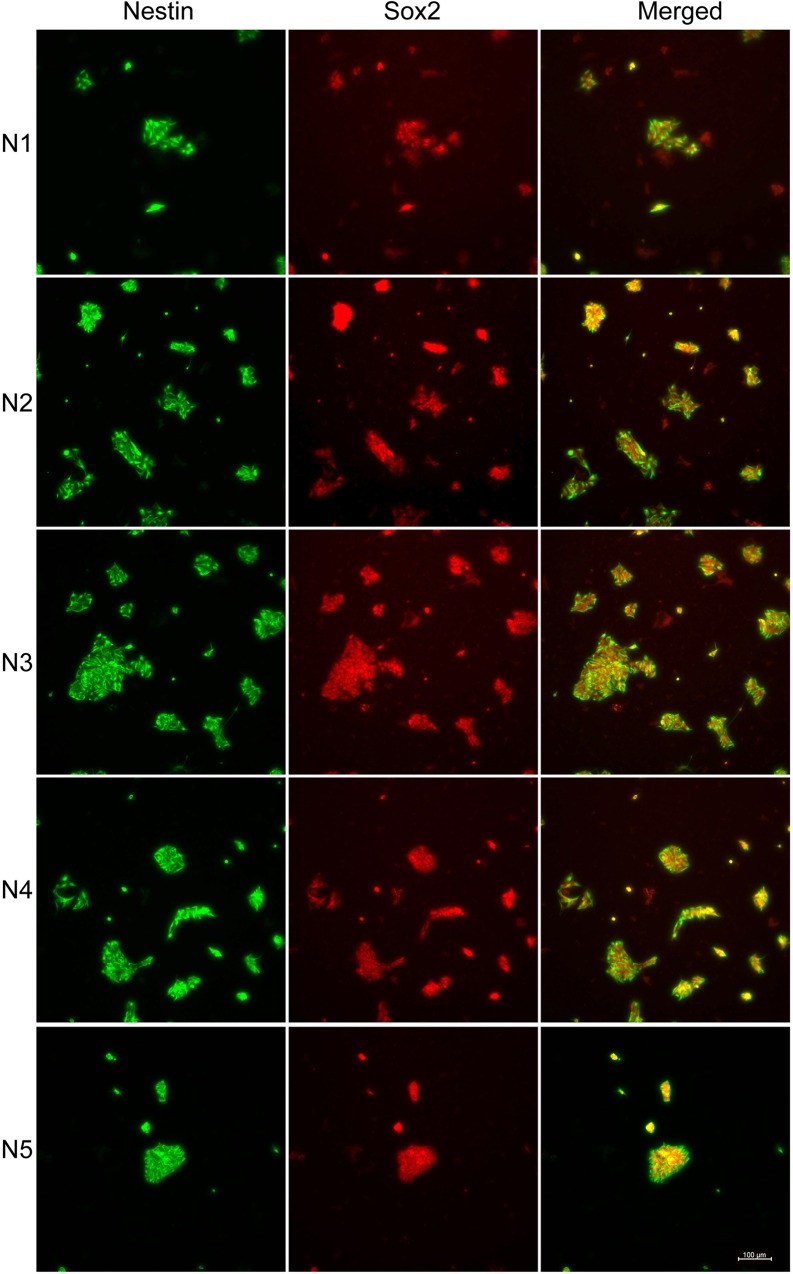
Characterization of neural stem cells by protein markers Nestin and Sox2. Induced pluripotent stem cell-derived neural stem cells were identified by different protein markers, Nestin (green) and Sox2 (red). All 5 AD patients’ iPSC-derived neural stem cells were Nestin- and Sox2-immunoreactive. The expression of both protein markers was higher in N3 and N4 and lower in N1 and N5. Merged images are illustrated in yellow. Scale bar: 100 μm.

**Fig 2:**
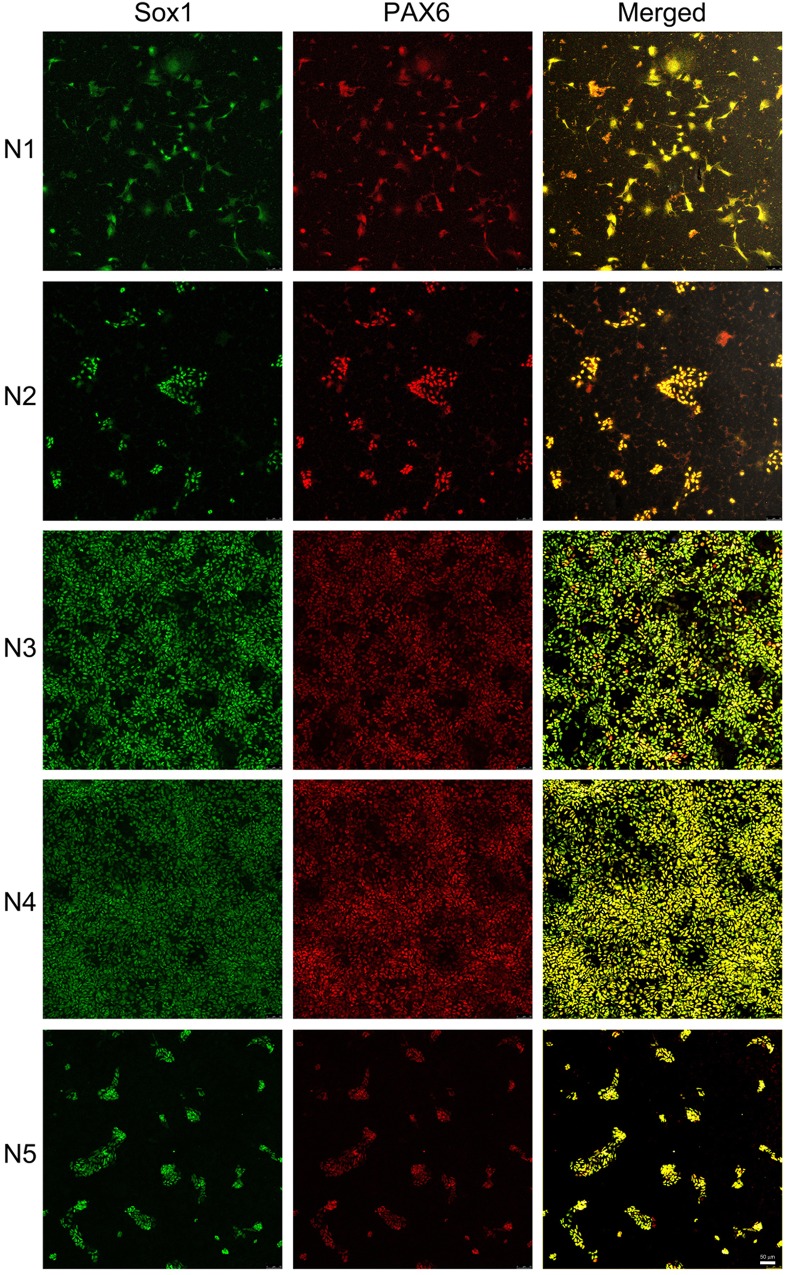
Characterization of neural stem cells by protein markers Sox1 and PAX6. iPSC-derived neural stem cells were identified by different protein markers, Sox1 (green) and PAX6 (red). Sox1 and PAX6 expression was higher in lines N3 and N4 compared to lines N1, N2 and N5. Merged images are illustrated in yellow. Scale bar: 50 μm.

iPSC lines from five AD subjects (AD1-AD5) were first induced to become neural stem cells (N1-N5, Fig 1). Neural stem cell lines (N1-5) were characterized by the expression of protein markers, Nestin and Sox2 (Fig 1), Sox1 and PAX6 (Fig 2). Nestin (green, Fig 1), a neuro-ectodermal stem cell marker, is a type VI intermediate filament protein that is expressed mostly in neural cells and is implicated in the growth of the axon [24, 25]. Sox2 (red, Fig 1) is a transcription factor that is essential for maintaining self-renewal, or pluripotency, of undifferentiated embryonic stem cells and has a critical role in maintenance of embryonic and neural stem cells [26, 27]. Sox1 and PAX6 (Fig 2) were found to be expressed in all five neural stem cultures. Sox1 is an activated neural stem/progenitor cell maker and transcription factor, and PAX6 controls the balance between neural stem cell self-renewal and neurogenesis [27–29].

**Fig 3:**
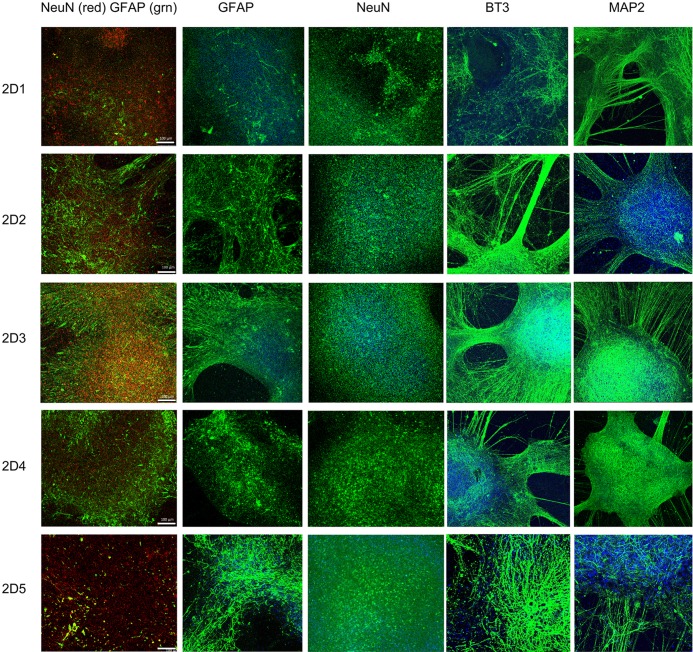
Characterization of 2D neuronal culture differentiated from neural stem cells. 2D neurons were immunostained with antibodies against the markers NeuN, GFAP, BT3 and MAP2. Differentiated neurons from all five subjects showed NeuN-, BT3- and MAP2-positive cells. Cells positive for the astrocyte marker GFAP were also presented. The left column of images is an illustration of merged NeuN (red) and GFAP (green) staining. NeuN (red) is highly expressed in the nucleus of differentiated neurons whereas GFAP (green) is expressed in the filament of the astrocytes. Nuclei staining was illustrated in blue across all images. Scale bar: 100 μm.

**Fig 4:**
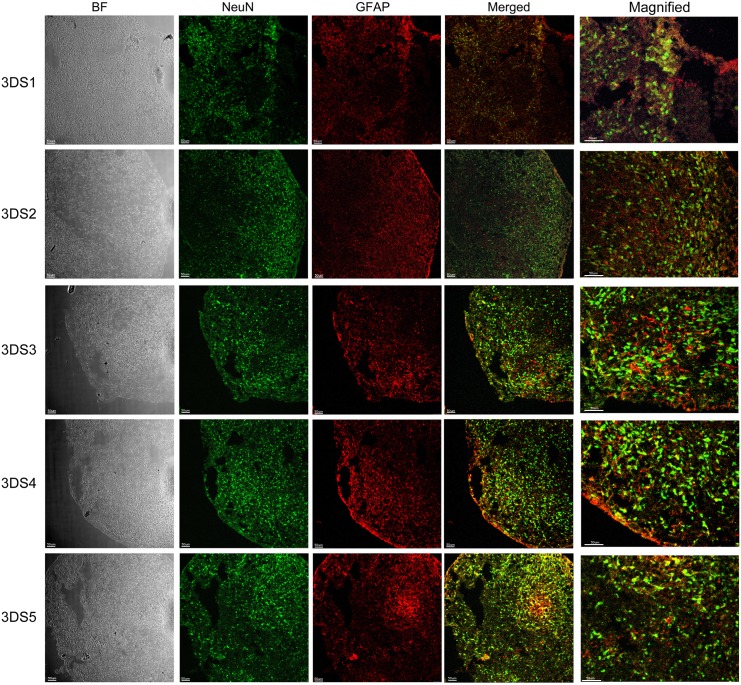
Characterization of 3D neuro-spheroid cultures by NeuN and GFAP. Neuro-spheroids (3DS1-5) were characterized by immunofluorescence staining of different protein markers, NeuN (green) and GFAP (red). Neuro-spheroids were positive for both NeuN and GFAP in all 5 cell lines. Merged and magnified images illustrate NeuN positive nucleus (green) and GFAP positive cells (red) localized in the filaments of astrocytes. Representative bright field (BF) images are presented on the left column. Scale bar: 50 μm.

**Fig 5:**
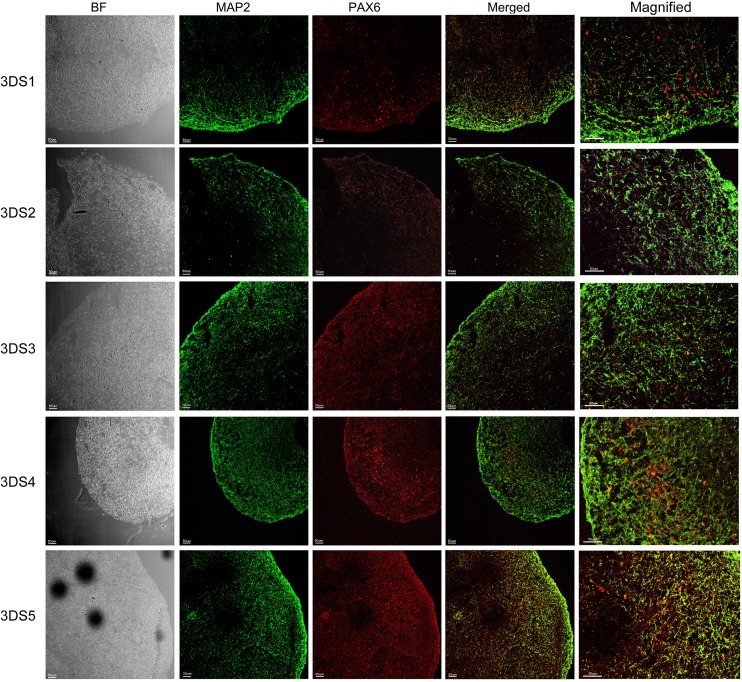
Characterization of 3D neuro-spheroid cultures by MAP2 and PAX6. Neuro-spheroids (3DS1-5) were characterized by immunofluorescence staining of different marker proteins MAP2 (green) and PAX6 (red). All cells are immunoreactive to neuronal marker MAP2. Because the neuro-spheroids were still undergoing differentiation at the time of the immunostaining, some cells were found immune-positive with early neuronal differentiation marker PAX6. Merged and magnified images showed different distribution of both cell types. Bright field (BF) images are represented in the left column. Scale bar: 50 μm.

We have further grown these neuronal stem cells in parallel into neuronal culture in two different environments, 2D neuronal culture (2D1-2D5, Fig 3) and 3D neuronal spheroids (3DS1-5, Figs 4 and 5) using a modified protocol [23]. To characterize these 2D and 3D neurons, we performed ICC to detect epitopes associated with immature and mature neurons. Confocal images from 2D neurons in five iPSC differentiated neuronal culture demonstrated protein marker expression of NeuN, GFAP, β tubulin III (BT3) and MAP2 (Fig 3). Serial sections from 3D neuronal spheroids (3DS1-3DS5; Figs 4 and 5, bright field (BF)) were stained with following antibodies: NeuN (green) and GFAP (red) (Fig 4), and MAP2 (green) and PAX6 (red; Fig 5). All of the sections from 3D neuronal spheroids exhibited staining patterns similar to that of the 2D cells (Fig 3). The presence of NeuN staining clearly indicated the withdrawal of the neurons from the cell cycle and the initiation of terminal differentiation of the neurons [30]. Almost all of neuronal stem cells were differentiated into neurons, as class III β-tubulin is a microtubule element expressed exclusively in neurons [31]. Our spheroids were also immunoreactive with GFAP antibody that we have used to stain human brain tissue in our previous studies [32], suggesting a mixed population of neurons and astrocytes/glia cells. MAP2 is an abundant neuronal cytoskeletal protein that binds to tubulin and associates with and stabilizes microtubules [33], and our differentiated neurons exhibited similar MAP2 staining. Expression of transcription factor PAX6, an early marker of neuronal differentiation [34], drove the differentiation of all five stem cell lines to neurons.

**Fig 6:**
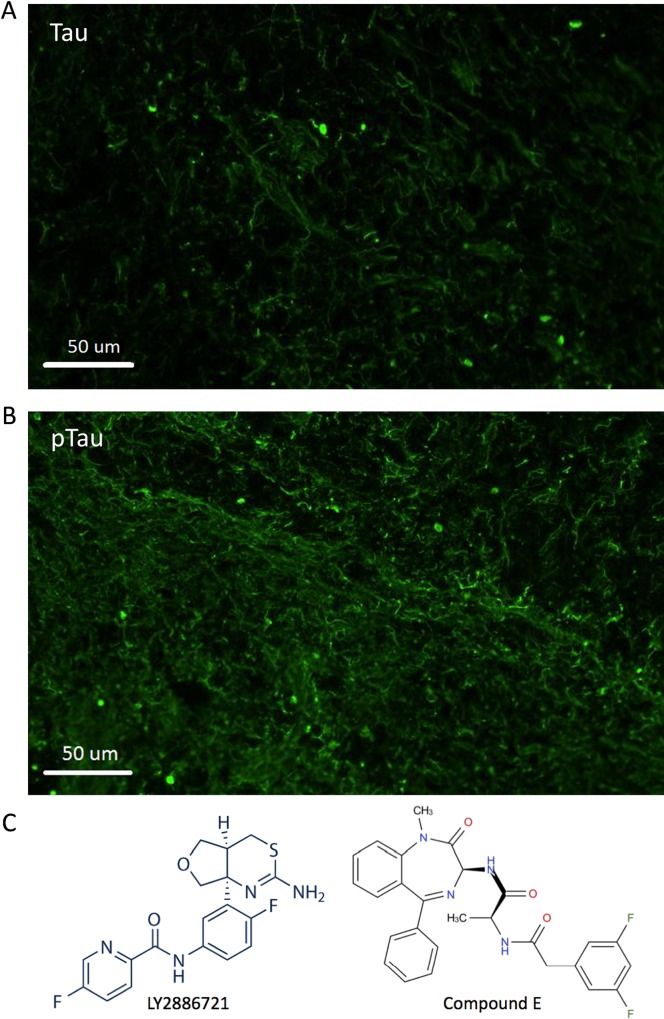
Structures of the BACE1 and γ-secretase inhibitors used to treat 3D neurons. A. Immunocytochemical staining of 3D neuro-spheroid section for Tau protein using antibody BT-2. B. ICC of neuro-spheroid section for phosphorylated Tau. Antibody AT270 specifically stains phosphor-Tau at residue Thr181. C. LY2886721, a potent and selective BACE1 inhibitor (left), and Compound E, a cell permeable non-competitive inhibitor of γ-secretase (right), are used to treat neurons.

We also characterized the 3D neuronal culture that has been known to recapitulate both amyloid β and Tau pathology [14]. In addition to Aβ quantification (see below), we performed ICC to detect Tau and phosphorylated Tau proteins using antibodies specifically targeting Tau (antibody BT-2; Fig 6A) or phosphorylated Tau at residue Thr 181 (antibody AT270; Fig 6B).

### Reduced Aβ40 & 42 production in 2D neurons treated with BACE1 or γ-secretase inhibitors

**Fig 7:**
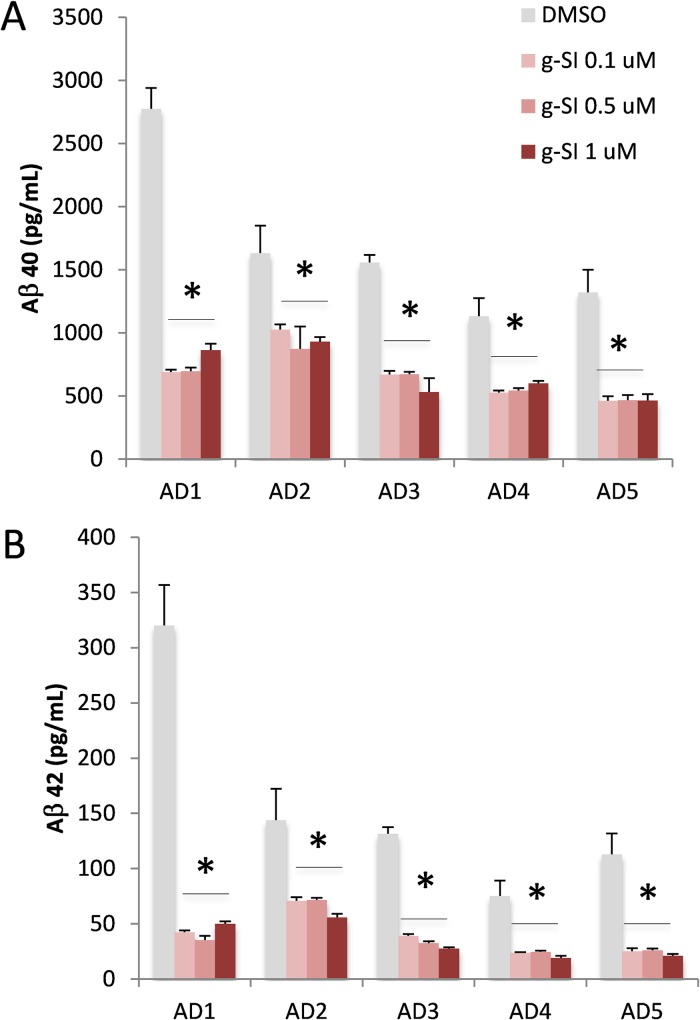
Treatment of 2D cell culture by a γ-secretase inhibitor. 2D neurons differentiated from five AD patients’ iPSC lines were treated with increasing concentrations of γ-secretase inhibitor (g-SI, 0.1, 0.5 and 1 μM; red) or DMSO as control (grey). A. Aβ40 levels in conditioned media from cells treated with γ-secretase inhibitor were significantly reduced. B. Aβ42 levels were significantly decreased in all 5 AD subjects after treatment with the γ-secretase inhibitor at all doses tested. The graph shows Mean±standard error of means (SEM); * represents p< 0.05, comparison of inhibitor vs. DMSO.

**Fig 8:**
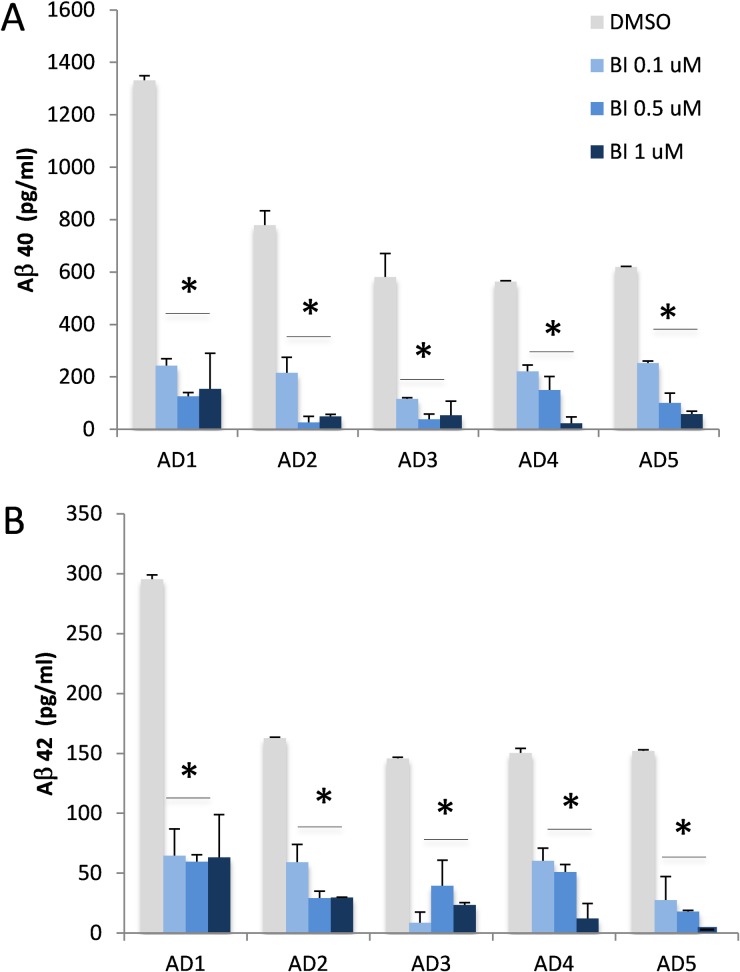
Treatment of 2D cell culture by a BACE1 inhibitor. 2D neurons differentiated from five AD patients’ iPSC lines were treated with increasing concentrations of BACE1 inhibitor (BI, 0.1, 0.5 and 1 μM; blue) or DMSO as control (grey) for two days. Conditioned media were collected and Aß 40 and 42 were quantified by ELISA. Both Aß 40 (A) and 42 (B) levels were significantly reduced in all lines from 5 AD subjects after the treatment with the BACE inhibitor at all doses tested. Levels of Aβ42 were undetectable in 1 μM BI-treated neurons derived from AD5. The graph shows Mean±SEM; * represents p< 0.05, comparison of inhibitor vs. DMSO.

After differentiation for 6–8 weeks, 2D neurons were treated with either BACE1 [35], γ-secretase inhibitor Compound E [36] (Fig 6C) or vehicle (DMSO), and conditioning media were collected for quantification of Aβ by ELISA [37]. γ-Secretase inhibitor Compound E is a widely used potent inhibitor for many in vitro and in vivo studies. The half maximal inhibitory concentration (IC_50_) of Compound E in most in vitro γ-secretase activity assays is in the low nM range [36]. When our 2D neurons were treated with 0.1 μM Compound E (g-SI, Fig 7), all neurons produced significantly less Aβ40 and Aβ42. Interestingly, the reduction of Aβ40 and Aβ42 did not increase when higher doses (up to 1 μM) of Compound E were used (Fig 7). The efficacy of BACE1 inhibitor was obvious in 2D neurons (Fig 8). When neurons were exposed to 0.1–1 μM BACE1 inhibitor (BI, Fig 8), a significant reduction of Aβ40 and 42 was observed in all five lines (Fig 8). All Aβ40 levels decreased dramatically when higher concentrations of BACE1 inhibitor were applied. A similar pattern was observed for Aβ42.

### Reduced Aβ40 & 42 production in 3D neurons treated with BACE1 or γ-secretase inhibitors

**Fig 9:**
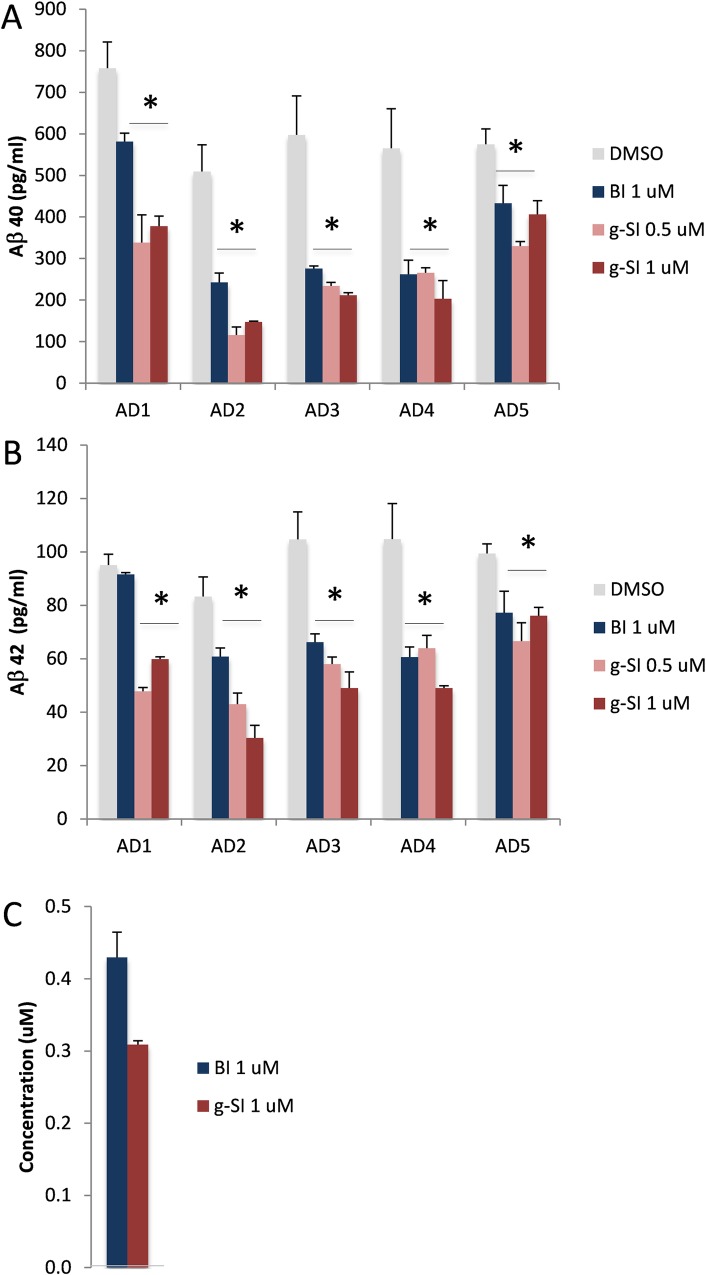
Treatment of 3D neuro-spheroids with BACE1 and γ-secretase inhibitors. 3D neuro-spheroids differentiated from five AD patients’ iPSC lines were treated with either BACE1 (BI, 1 μM; blue) or γ-secretase inhibitor (g-SI, 0.5 or 1 μM; red) for two days. Media were collected for Aß 40 (top) and 42 (middle) ELISA quantification. A. Lower levels of Aß 40 were found in media after treatment with either BACE1 or γ-secretase inhibitors. B. Aß42 levels from AD1 remained unchanged after being exposed to BACE1 inhibitor. Aβ42 levels from other AD subjects were reduced. C. The drug levels of BACE1 inhibitor (blue, dosing at 1 μM) and γ-secretase inhibitor (red, dosing at 1 μM) in 3D neuro-spheroids were quantified by LC-MS/MS. The graph shows Mean±SEM; * represents p< 0.05, comparison of inhibitor vs. DMSO.

After 9 weeks of differentiation, 3D neuronal spheroids were treated with BACE1 or γ-secretase inhibitor for two days and media was collected for Aβ 40 and 42 measurements by ELISA. Treating the cells for two consecutive days markedly decreased both Aβ 40 and 42 (Fig 9A and 9B), which was significant in all cases except for Aβ42 from the AD1 neurons treated with BACE1 inhibitor (Fig 9B). 3D neurons derived from subject AD1 did not exhibit reduced Aβ42 production in the presence of BACE1 inhibitor, unlike all of the remaining four lines (AD2-5) that exhibited significantly less Aβ42.

We found that neurons from five AD patients generated similar levels of Aβ40 and Aβ42 in the absence of any compounds (Fig 9). Although both BACE1 and γ-secretase inhibitors are extremely potent in previously published studies using stable mammalian cell lines, primary 2D mouse neuronal culture, and in vitro enzymatic activity assays [35, 36], our 3D neuro-spheroids seemed to be responding less to these potent inhibitors. Interestingly, the efficacies of the same compounds in two different systems, 2D versus 3D, were quite different. 3D neuro-spheroids showed less reduction of Aβ compared to 2D neurons in the presence of the same concentrations of BACE1 or γ-secretase inhibitors (Fig 9 vs. Figs 7 and 8).

One possibility for this discrepancy is the bioavailability of the inhibitors. In the 2D environment, all neurons are exposed to the same concentration of inhibitors evenly; in 3D environment, surface neurons within each spheroid may be exposed to higher drug concentrations than internal cells. To determine whether these two inhibitors were permeable to neuro-spheroids, we collected the same number of neuronal spheroids exposed to BACE1 or γ-secretase inhibitor and extracted drugs for LC-MS/MS quantification (Fig 9C). We found that these compounds accumulated and remained inside of neuro-spheroids at ~30% (γ-secretase inhibitor, Fig 9C, red) to ~40% (BACE1 inhibitor, Fig 9, blue) of dosing concentration, suggesting that the reduction of drug efficacy was related to decreased exposure to drugs.

### Proteomic analysis of 3D neurons reveals molecular signatures that affect inhibitor efficacy

To understand the possible cause that rendered reduced efficacy of BACE1 inhibitor in 3D neuronal culture derived from the subject AD1, we prepared lysates from 3D neurons and subjected them to proteomic analysis using Mass Spectrometry. We analyzed these samples by labelling tryptic peptides with TMT 6-plex reagents. The relative levels of several gene products were calculated; since the efficacy of BACE1 inhibitor was reduced in AD1, we compared individual subjects to subject AD1 (Table 2).

**Table 2:** Relative protein levels in 3D neurons differentiated from five AD subject-derived iPSC lines.

Accession	Description	AD1	AD2	AD3	AD4	AD5
P05067	Amyloid β A4 precursor protein	1.00	0.94	0.63	0.58	0.71
Q16143	β-synuclein	1.00	1.24	1.39	1.47	0.88
O60641	Clathrin coat assembly protein AP180	1.00	1.38	0.61	0.56	0.63
Q00610	Clathrin heavy chain 1	1.00	0.94	0.60	0.53	0.61
P09496	Clathrin light chain A	1.00	0.96	0.64	0.56	0.67
P09497	Clathrin light chain B	1.00	1.02	0.58	0.52	0.58
O76070	γ-synuclein	1.00	0.87	0.58	0.59	0.67
P49840	Glycogen synthase kinase-3α	1.00	1.38	0.64	0.58	0.63
P49841	Glycogen synthase kinase-3β	1.00	1.04	0.71	0.57	0.64
P10636	Microtubule-associated protein tau	1.00	1.54	1.34	0.61	0.96
P07196	Neurofilament light polypeptide	1.00	1.78	0.58	0.67	0.70
P07197	Neurofilament medium polypeptide	1.00	1.78	0.60	0.59	0.70

We found that the average levels of the BACE1 substrate APP in other subjects were reduced compared to levels in subject AD1. There was a minor reduction of APP in subject AD2 but AD3, AD4 and AD5-derived 3D neurons expressed significantly less APP. The levels of APP were decreased 30–40% in these subjects compared to AD1. With a reduction of substrate APP, the efficacy of BACE1 inhibitor in these neuronal lines was better than in AD1.

We also found that levels of clathrin heavy and light chains were similarly reduced in these lines, compared to AD1. An average of 40% reduction was observed in subjects AD3, AD4 and AD5, with insignificant reduction found in subject AD2. Clathrin and its partner Adaptor Protein 2 are involved in endocytosis of APP and its C-terminal fragments [38, 39]. A reduction of clathrin and related proteins likely decreased the level of APP to interact with BACE1 and subsequent cleavage for Aβ production. When the BACE1 inhibitor was present, its efficacy was more obvious in those lines compared to AD1 (Fig 9B).

## Discussion

Drug screening using iPSC-differentiated cells is a promising approach to evaluating potential therapeutic drugs. Neuronal culture is probably a more physiologically relevant assay system than stable mammalian cell lines, though much work remains to develop and characterize this system. To date, most cell-based assays have employed single layer cell cultures for testing compounds. A recently described 3D neuronal culture system provides a novel cellular model for evaluation of drug efficacy [14]. In this study, we combined these two technical platforms, iPSC-differentiated cells and 3D neuro-spheroid, to address several challenges encountered during drug screening and development.

In AD research, drug development has been slowed by the variable pharmacology of drugs in stable cell lines, primary cultures, and animal models. In general, primary neuronal culture is a preferred cellular model for testing drugs. In our study, all 2D neuronal cultures exhibited significant reduction of Aβ40 and Aβ42 when cells were exposed to BACE1 or γ-secretase inhibitor (Figs 7 and 8). However, inhibition of Aβ production was quite variable amongst our iPSC-derived neuronal lines, with some neuronal cultures exhibiting minimal response to standard BACE1 inhibitor and most exhibiting saturation of inhibition (Figs 7 and 8). The former phenomenon may be related to individual genotypes, and suggests that Aβ generation and turnover may be affected by individual genetic background, an observation with significant implications for development of Aβ-directed AD therapeutics. Our research subjects have not been genetically evaluated, and future research will focus on identification of genotypes associated with this variation. One specific study is underway to understand the apoE genotypes that might be different among these five subjects.

The latter phenomenon, saturation of inhibition, may be related to variable drug bioavailability, drug metabolism, or cellular responsivity. Our present data do not shed any light on the mechanism of saturation in some cell lines. We note that saturation could be related to the same mechanisms underlying variable response to inhibitors. Either way, these data indicate that evaluating dose-responses of candidate anti-amyloid therapeutics may require individualized testing based on cellular responses or yet-to-be determined genetic markers. Identification of single-nucleotide polymorphism (SNP) that correlates with cellular responses may provide important mechanistic and clinical information.

In this study, we found a significant reduction of Aβ40 and Aβ42 in the conditioned media of 3D neuro-spheroids exposed to BACE1 or γ-secretase inhibitor. This finding is consistent with findings in 2D cultures, indicating that the direction of the drug effect is identical under 2D and 3D conditions, as expected. However, the magnitude of reduction in 3D cultures was less than that observed in 2D neurons exposed to the same concentration of inhibitors. We quantified drug exposure of 3D neuronal spheroids after two-day of treatment and found it to be less than the dosing concentrations (Fig 9). We reasoned that neurons enclosed inside of spheroids had less overall exposure to drugs. During the early stages of treatment, before the concentration of compounds inside and outside of spheroids reach equilibrium, significant amounts of Aβ may have been generated inside the spheroids and eventually released to the media. The alternative interpretation of these data, that 3D configuration of neurons alters their response to drugs, is contradicted by our result showing the same direction of drug effect and is not supported by any known example of altered cell physiology based on 3D vs. 2D cellular configuration. We therefor conclude that the reduced efficacy of BACE1 and γ-secretase inhibitors in 3D configurations is related to reduced drug exposure of cells within the spheroids, an interpretation consistent with our direct measurements of drug concentrations (Fig 9C). While this result may not be unexpected, the difference is substantial even on a microscopic scale over the course of 2 days of treatment.

Our findings are relevant to the design of future screening protocols using 3D spheroids compared to 2D neuronal cultures. 3D neuronal culture has a number of advantages and disadvantages compared to 2D neuronal culture. The first disadvantage of 3D neurons is the reduced diffusion of candidate drugs. Even across the tiny (10 um) distance between the peripheral and internal regions of iPSC-derived neuro-spheroids, differences in concentration exist. Identifying diffusion-limiting factors of 3D neuronal cultures may help to advance diverse areas of neurotherapeutics and the consistency needed for drug screening. Chronic dosing may represent a second disadvantage to high throughput screening using 3D cultures. The increased time requirement for uniform bioavailability within the 3D spheroids indicates that additional development is needed before this approach is ready for large-scale drug screening.

The advantages of using iPSC-differentiated 3D neuronal system for drug screening are likely offset the disadvantages. First, the 3D environment offers some anatomical similarities to mature brain (compared to 2D cultures). The 3D cells better represent the native target of the drug. Whether a 2D configuration is associated with any differences in cell physiology is unknown, and using 3D systems avoids this uncertainty. Second, 3D cultures allow for microscopic evaluation of spatial features related to drug effects in a system that more closely resembles the target tissue. It is certainly possible, if not likely, that cytoskeletal dynamics, such as Tau binding to microtubules, is related to neuronal spatial configuration. Third, our 3D cell system allows us to quantify drug levels that is not available in our traditional 2D assay system. Since the introduction of 3D neuronal culture for Aβ and Tau quantification [14], few studies have utilized this system to evaluate drug efficacy, and there is no report on drug bioavailability in 3D neurons. The physical properties of 3D neuro-spheroids allow LC-MS/MS based quantification of drug exposure and assessment of dose-dependent drug efficacy.

The uniqueness of using iPSC-differentiated neurons is obvious by the variation among the five lines that we tested. We found that 3D neuro-spheroids differentiated from subject AD1 did not respond to BACE1 inhibitor such that no reduction of Aβ42 was observed. This is an unexpected finding with implications for both the biology of APP processing and the clinical application of secretase inhibitors. If the same class of BACE1 inhibitors repetitively shows a lack of efficacy in blocking Aβ production in a number of subjects, we probably would not enroll these subjects for clinical trials for the BACE1 inhibitors. Analysis of these lines for SNP may yield highly useful pharmacogenetic markers for individualization of treatment strategies for amyloid reduction. The molecular mechanism behind this phenomenon is obscure. One possible explanation is that genetic variation, not necessary familial AD mutations like Swedish mutation at the BACE1 cleavage site [40], increases the expression levels of several genes like APP and BACE1. High expression of substrate and enzymatic activity may compensate for the reduction by BACE1 inhibitors. Similarly, levels of clathrin proteins were higher in neurons from AD1 subject (Table 2), indicating a better endocytic process for APP and its cleavage by BACE1 for Aβ generation. Our MS-based proteomic analysis of all 3D neuronal culture provides many proteins that were up/down-regulated across five lines, presenting a venue to understand the individual profile of drug pharmacology using systems biology.

The individual variation may also relate to differential Aβ clearance in 2D and 3D systems. Compared to four subjects AD2-5, we found relatively higher Aβ levels in 2D neuronal culture derived from subject AD1 (Fig 7); however, this difference was diminished in 3D culture (Fig 9). While we do not have a clear understanding of this difference, this phenomenon could be related to Aβ clearance. In 2D neurons, Aβ clearance may be reduced in subject AD1, leading to higher levels of Aβ remaining in the system. Aβ clearance among five 3D neuronal lines might be similar, and no significant difference could be observed. Such cell functional variations between 2D and 3D neuronal culture are critical pieces of the puzzle; linking them together will improve our understanding of the outcomes of drug trials in individual-derived neuronal cultures and support the development of pharmacogenetic markers for AD treatment. Our pilot study included only five subjects, and additional subjects will be required to generate sufficient power to probe for genetic markers. Nevertheless, the methodology we developed here can be readily expanded.

Future studies are needed to understand the cell biology of 3D neuro-spheroids derived from iPSC and their response to therapeutic applications. The 3D model system is ideal for revealing cell-cell interaction and communication, and it is also important to understand the interaction among different cell types, i.e., neurons, microglia, and astrocytes. Intracellular protein trafficking plays an important role in responding to drug treatment, and 3D neuro-spheroid provides an excellent model to reveal subcellular activities in the native 3D configuration. Such studies represent a unique opportunity to dissect the molecular variation in AD subjects and develop clinically useful markers for individualized treatment.
